# Long Non-coding RNA LRNA9884 Promotes Acute Kidney Injury via Regulating NF-kB-Mediated Transcriptional Activation of MIF

**DOI:** 10.3389/fphys.2020.590027

**Published:** 2020-10-29

**Authors:** Yingying Zhang, Patrick Ming-Kuen Tang, Yangyang Niu, Cristina Alexandra García Córdoba, Xiao-Ru Huang, Chen Yu, Hui-Yao Lan

**Affiliations:** ^1^Department of Nephrology, Tongji Hospital, Tongji University School of Medicine, Shanghai, China; ^2^Department of Anatomical and Cellular Pathology, State Key Laboratory of Translational Oncology, Prince of Wales Hospital, The Chinese University of Hong Kong, Shatin, Hong Kong; ^3^Department of Medicine & Therapeutics, Li Ka Shing Institute of Health Sciences, Lui Che Woo Institute of Innovative Medicine, Shenzhen Research Institute, The Chinese University of Hong Kong, Shatin, Hong Kong; ^4^Guangdong-Hong Kong Joint Laboratory on Immunological and Genetic Kidney Diseases, Guangdong Academy of Medical Sciences, Guangdong Provincial People’s Hospital, Guangzhou, The Chinese University of Hong Kong, Shatin, Hong Kong

**Keywords:** lncRNA, inflammation, AKI, NF-κB, macrophage migration inhibitory factor

## Abstract

Acute kidney injury (AKI) is one of the most common complications affecting hospitalized patients associated with an extremely high mortality rate. However, the underlying pathogenesis of AKI remains unclear that largely limits its effective management in clinic. Increasing evidence demonstrated the importance of long non-coding RNAs (lncRNAs) in the pathogenesis of AKI, because of their regulatory roles in transcription, translation, chromatin modification, and cellular organization. Here, we reported a new role of LRNA9884 in AKI. Using experimental cisplatin-induced AKI model, we found that LRNA9884 was markedly up-regulated in the nucleus of renal tubular epithelium in mice with AKI. We found that silencing of LRNA9884 effectively inhibited the production of inflammatory cytokines MCP-1, IL-6, and TNF-α in the mouse renal tubular epithelial cells (mTECs) under IL-1β stimulation *in vitro*. Mechanistically, LRNA9884 was involved into NF-κB-mediated inflammatory cytokines production especially on macrophage migration inhibitory factor (MIF). Collectedly, our study suggested LRNA9884 promoted MIF-triggered the production of inflammatory cytokines via NF-κB pathway after AKI injury. This study uncovered LRNA9884 has an adverse impact in AKI, and targeting LRNA9884 might represent a potential therapeutic target for AKI.

## Introduction

Acute kidney injury (AKI) is defined as a sudden deterioration in kidney function over a short period of time. Studies have reported that 3.2–21% of hospitalized patients and up to 50% of patients admitted to intensive care units develop AKI, with a mortality rate ranging from 40 to 60%. If there are other comorbidities with AKI, the incidence and mortality rates can be as high as 30 and 80%, respectively (Varrier et al., 2015; Agarwal et al., 2016). Studies found that AKI significantly prolonged the length of a patient’s stay in hospital and also significantly increased overall inpatient expenses (about $24 billion annually in the United States) (Silver et al., 2017). The cost required to treat AKI is comparable to treating other serious conditions such as stroke, pancreatitis and pneumonia. Taken together, these findings highlight the need for new therapies to treat this serious condition.

Long non-coding RNAs (lncRNAs) are a class of RNA molecules longer than 200 nucleotides but lacking protein-coding potential. These are transcribed from the mammalian genome and have emerged as important regulators of transcription, translation, chromatin modification and cellular organization. Compared to protein coding RNAs, lncRNAs have higher specificity to disease conditions, making them promising diagnostic and prognostic biomarkers as well as therapeutic targets. Our previous studies demonstrated that lncRNA (Erbb4-IR) induced renal fibrosis via Smad3-Smad7 pathway in the mouse UUO-induced kidneys (Feng et al., 2018), nevertheless, under diabetic conditions Erbb4-IR improved renal inflammation via miR-29b (Sun et al., 2018). Meanwhile, increasing evidence suggested that lncRNAs may be a potential target for treatment of acute and chronic kidney diseases (Mercer et al., 2009; Tang et al., 2018b). Kato et al. (2016) reported that a chemically modified oligonucleotide targeting lnc-MGC inhibited cluster microRNAs, glomerular extracellular matrix (ECM) and hypertrophy in the early stages of diabetic nephropathy. Li et al. (2017) demonstrated that the expression of lncRNA MALAT1 antagonized the inhibitory effect of miR-23c on hyperglycemia-induced cell pyroptosis in HK2 cells. However, the functional and pathogenic roles of lncRNAs in kidney diseases are still largely unclear and remain to be further elucidated.

Our previous study identified 21 novel Smad3-dependent lncRNAs were participated in renal inflammation and fibrosis from experimental mouse kidney disease models (Zhou et al., 2014). Among them, we demonstrated that targeting Erbb4-IR may represent a novel therapy for inhibiting progression of renal fibrosis (Feng et al., 2018; Sun et al., 2018), whereas Arid-IR may related to the renal inflammation (Zhou et al., 2015). Recently, we further uncovered that LRNA9884, one of the 21 Smad3-dependent lncRNAs, was involved in chronic diabetic kidney injury of db/db mice (Zhang et al., 2019b). Nevertheless, its implication in AKI is still unknown. Therefore, in the present study we further explored the potential role of LRNA9884 in AKI by using our clinical-related cisplatin-induced AKI mouse model (Lv et al., 2017; Li et al., 2018a). Interestingly, we found that LRNA9884 was evoked in the cisplatin-injured in mice especially on the nucleus of renal tubular epithelium. Mechanistically, we found that inflammatory cytokine IL-1β was capable for triggering LRNA9884 expression in the mouse renal tubular epithelial cells (mTECs) *in vitro*. More importantly, we finally identified the essentialness of LRNA9884 in NF-kB-mediated renal inflammation by regulating the production of pathogenic effector macrophage migration inhibitory factor (MIF) at genomic level. Thus, LRNA9884 may represent as a potential therapeutic target for AKI management.

## Materials and Methods

### Animal Model

C57BL/6J mice were inoculated with cisplatin at a dose of 20 mg/kg (Sigma-Aldrich, St. Louis, MO, United States) by intraperitoneal injection to develop an experimental model of AKI. All mice were sacrificed by intraperitoneal injection of ketamine/xylene. To verify the model, the kidneys were harvested 3 days after cisplatin treatment and were subsequently processed for hematoxylin-eosin (H&E) and Periodic Acid Schiff (PAS) staining for histological studies. All studies were approved by the Animal Experimentation Ethics Committee of The Chinese University of Hong Kong and the experimental protocols were carried out in accordance with approved guidelines (Lv et al., 2017; Li et al., 2018a).

### Cell Culture

Immortalized murine renal proximal tubular epithelial cells (mTECs) were cultured in DMEM/F-12 (Gibco, Carlsbad, CA, United States), supplemented with 10% FBS (Gibco) and 1% antibiotic/antimycotic solution (Life Technologies, Grand Island, NY, United States). mTECs, a gift from Dr. Jeffrey B. Kopp, NIH, is immortalized murine kidney proximal tubular epithelial cells. To determine if LRNA9884 expression could be induced *in vitro*, mTECs were stimulated with inflammation inducing factors such as IL-1β (10 μg/ml; R&D Systems, Minneapolis, MN, United States), TNF-α (10 ng/ml; R&D Systems, Minneapolis, MN, United States), LPS (100 ng/ml; L2630, Sigma-Aldrich, United States), C reactive protein (CRP) (10 μg/ml; source), and Transforming Growth Factor β (TGF-β) (5 ng/ml, source) at 0.5 h. Subsequently, these samples were employed to examine LRNA9884 expression using real-time PCR (Tang et al., 2018a; Zhang et al., 2019b). To inhibit NF-κB activity, cells were pretreated with the NF-κB inhibitor Bay 11-7085 at a dose of 10 μM, (sc-202490; Santa Cruz Biotechnology, Santa Cruz, CA, United States) for 2 h before Il-1β stimulation.

### *In situ* Hybridization

LRNA9884 expression in the AKI kidney was detected using *in situ* hybridization (ISH), as previously described (Feng et al., 2018; Sun et al., 2018; Zhang et al., 2019b). After fixation in 4% (w/v) paraformaldehyde with 1% (v/v) DMSO, the kidney sections were rehydrated, permeabilised, pre-hybridized, and eventually hybridized. During the last phase, the sections were hybridized with a locked nucleic acid–digoxigenin labeled LRNA9884 probe (5′-ACTTGAAGGGTCCAGAAGAGAT-3′) (Exiqon, Vedbaek, Denmark) or negative control scramble probe (5′-GTGTAACACGTCTATACGCCCA-3′) (Exiqon). The sections were incubated with anti-digoxigenin antibody (11093274910, Roche Diagnostics, Indianapolis, IN, United States) conjugated to alkaline phosphatase and developed with phosphate/nitroblue tetrazolium (SigmaAldrich, St. Louis, MO, United States).

### Fluorescence *in situ* Hybridization and Immunofluorescence Assay

The cells and the kidney sections were fixed with 4% (w/v) paraformaldehyde, washed three times with 1 × PBS for 5 min, and washed once with distilled water. Prehybridization was performed at 37°C for 4 h in an incubator. The concentration of the hybrid solution were set with different gradients (5, 10, 50, and 100 μM) of probes. Hybridization was conducted overnight at 42°C in the incubator. After hybridization, sections were washed by gradient SSC, dripped with DAPI and sealed. All the equipment were disinfected with DEPC water. The fluorescence *in situ* hybridization (FISH) kit used in this method was purchased from Guangzhou Ruibo (C10910; RiboBio), the probe sequence was identical to the *in situ* hybridization probe, preceded by cy3-fluorescence labeling (Sun et al., 2018).

### Western Blotting Analysis

Protein from the renal tubular epithelium was extracted and western blot analysis was performed as previously described (Tang et al., 2017, 2018c; Li et al., 2020). The antibodies used in this study included phospho- NF-κB/p65 (Ser536, CST), NF-κB/p65 (CST), TNF-α, IL-1β, MCP-1, β-actin (Santa Cruz), and LI-COR IRDye 800-labeled secondary antibodies (Rockland Immuno-chemicals). The detection of specific signals was performed employing Odyssey infrared imaging system (LI-COR Biosciences, Lincoln, NE, United States) and quantified using Image J software (National Institutes of Health)^1^. The ratio for the protein detected was normalized against β-actin and the results were expressed as the mean ± standard error of the mean ± (SEM) (Wang et al., 2018).

### RNA Extraction and Quantitative Real-Time PCR

Total RNA was extracted from cells and real-time PCR was performed using an Option 2 instrument (Bio-Rad, Hercules, CA, United States) with IQ SYBR green supermix reagent (Bio-Rad, Hercules, CA, United States). The primers used in this study were mouse mRNA MCP-1, IL-1β, TNF-α, LRNA9884 and MIF as described in Supplementary Table 1. β-actin housekeeping gene was used as the internal control. Results were expressed by fold changes to controls as individual dots pattern.

### Enzyme-Linked Immunosorbent Assay

We collected medium from stimulated mTEC cells to detect inflammatory and pro-inflammatory cytokines production by using an enzyme-linked immunosorbent assay kit. TNF-a, MCP-1, and IL-6 were measured with a Quantikine ELISA Kit (R&D Systems) according to the product protocols (Zhang et al., 2019a).

### Statistical Analysis

All the data are expressed as the mean ± SEM. Statistical analyses were performed using two-way analysis of variance as appropriate. Tests were performed with GraphPad Prism 5 (GraphPad Software, La Jolla, CA, United States). A *P*-value <0.05 was considered to be a significant finding.

## Results

### LRNA9884 Is Highly Expressed in the Cisplatin-Induced AKI *in vivo*

**FIGURE 1:**
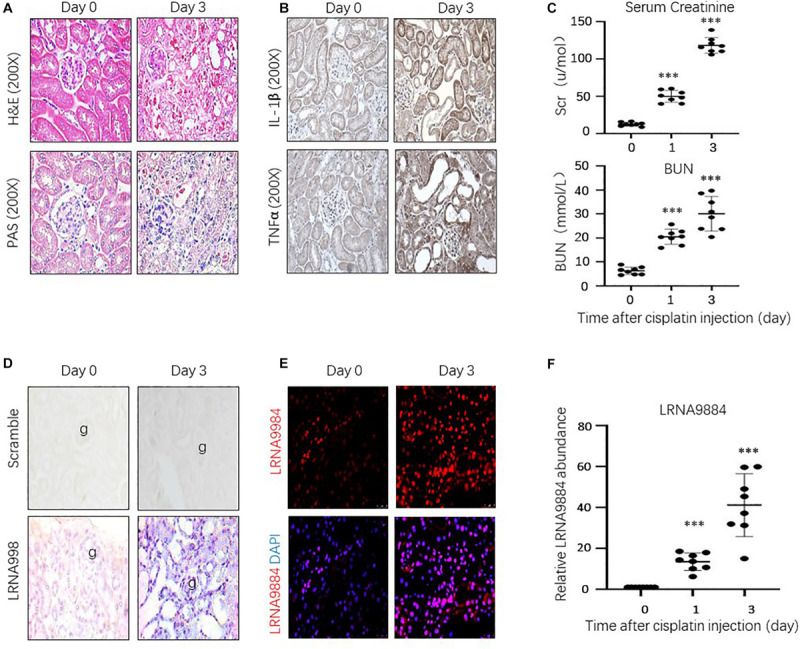
LRNA9884 and inflammatory cytokine expression in mice with cisplatin-induced AKI. **(A)** H&E staining and PAS staining shows changes in renal histology after AKI injury at day 0 and day 3. **(B)** Immunohistochemistry (IHC) of IL-1β and TNF-α expression (200× magnification). **(C)** Serum creatinine and blood urea nitrogen (BUN) of AKI mice. **(D,E)** ISH and FISH detects LRNA9884 expression at day 0, and day 3 after AKI injury (200× magnification). **(F)** Real-time PCR shows expression of LRNA9884 in mice kidneys. Bar represents the mean ± SEM for groups of eight mice. ***P < 0.001 vs. mice at day 0.

The experimental model of AKI was developed using C57BL/6J mice treated with cisplatin (20 mg/kg) (Lv et al., 2017). Histological examination revealed loss of brush border of renal epithelial cells, lumen dilation of renal tubular system, and cytoplasmic vacuolar degeneration and necrosis of tubular epithelium in the AKI model compared to the control group. Serum creatinine and urea nitrogen were increased in the AKI mice form day 1. These results indicated that the AKI model was successfully implemented (Figures 1A,C). Inflammatory cytokines were up-regulated in the AKI kidneys such as IL-1β and TNFα, indicated that activating inflammatory signals involved in cisplatin-induced AKI (Figure 1B). Furthermore, *ISH* and *FISH* examination of renal tubular epithelial cells harvested from the cisplatin-induced AKI model showed that expression levels of LRNA9884 increased gradually with AKI progression. Moreover, it indicated that LRNA9884 expression was mainly located in the nuclei of renal tubular epithelial cells (Figures 1D,E). Quantitative real-time PCR analysis of LRNA9884 expression revealed that AKI mice models had a greater intra-nuclear amount of LRNA9884 compared to the control group on the first day after cisplatin injection (*P* < 0.001). Additionally, the LRNA9884 expression on the third day was greater than on day one (*P* < 0.001) (Figure 1F). These results demonstrated that LRNA9884 expression was elevated and localized in the nuclei of tubular epithelia cells from the AKI kidney.

### LRNA9884 Is a Positive Feedback to the IL-1β Driven Renal Inflammation *in vitro*

**FIGURE 2:**
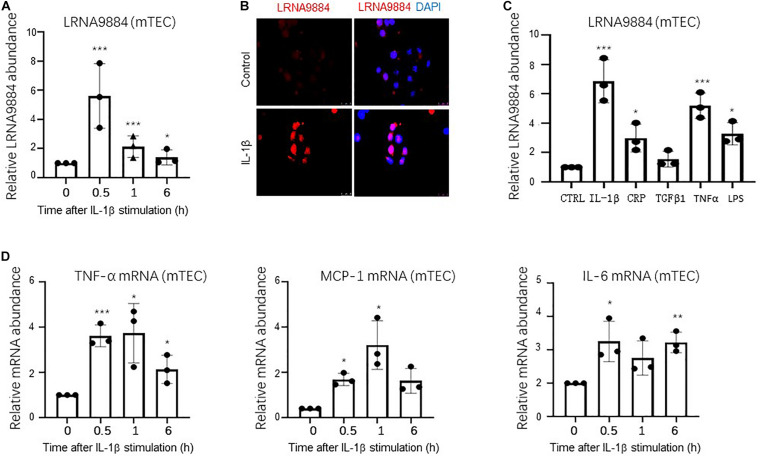
IL-1β triggers LRNA9884 and inflammation cytokines in mTECs. **(A)** Real-time PCR showed that IL-1β (10 ng/ml) induced LRNA9884 expression in mTECs in a dose-dependent manner. **(B)** FISH assay detected an increase in LRNA9884 expression after IL-1β treatment, primarily localized in the nucleus of mTECs. **(C)** Real-time PCR shows LRNA9884 expression after IL-1β, CRP, TGF-β, TNFα, and LPS treatment in mTECs. **(D)** Real-time PCR shows TNF-α, MCP-1 and IL-6 expression after IL-1β treatment. Bars represent the mean ± SEM for at least three independent experiments. *P < 0.05, **P < 0.01, ***P < 0.001 vs. time 0 h.

**FIGURE 3:**
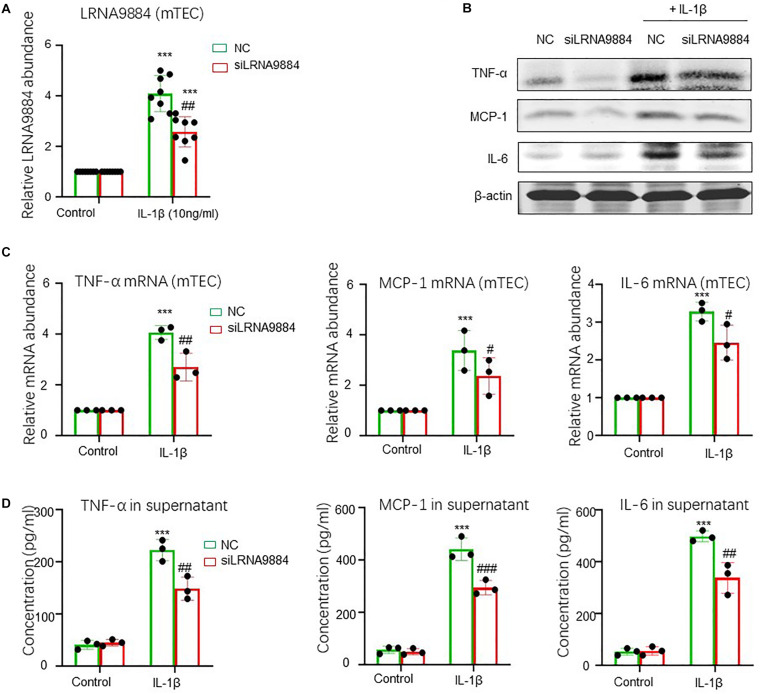
Silencing of LRNA9884 blocks inflammatory cytokine expression. **(A)** Real-time PCR results shows LRNA9884 expression level for siLRNA9884 and nonsense-treated control (NC) after IL-1β-induced in mTECs. **(B)** Western blot shows expression of TNF-α, MCP-1 and IL-6 in IL-1β-induced mTECs. **(C)** Real-time PCR shows TNF-α, MCP-1 and IL-6 expression in NC and siLRNA9884 mTECs for control and IL-1β groups. Bars represent the mean ± SEM for at least three independent experiments. **(D)** ELISA shows TNF-α, MCP-1 and IL-6 expression in NC and siLRNA9884 mTECs for control and IL-1β groups. ***P < 0.001 vs. Control group, ^#^*P* < 0.05, ^##^*P* < 0.01, ^###^*P* < 0.001 vs. NC group.

*In vitro* analysis of cultured mTECs stimulated with IL-1β showed that LRNA9884 expression increased significantly after IL-1β (10 ng/ml) stimulation for half hour (*P* < 0.001) (Figure 2A). Moreover, FISH further indicated that the IL-1β induced LRNA9884 was predominantly located in the nucleus (Figure 2B), which was consistent with the *in vivo* results obtained from ISH (Figure 1B). It was further explored whether other stimuli besides IL-1β could induce an increase in the expression of LRNA9884. Interestingly, our results showed that TNF-α, LPS, CRP, and TGF-β could also trigger LRNA9884 expression compared to the negative control group (CTRL) (Figure 2C), but IL-1β was the greatest extend (*P* < 0.001). In addition, we found that IL-1β induced a high expression of various inflammatory factors such as tumor necrosis factor (TNF-α), monocyte chemotactic protein (MCP-1) and interleukin 6 (IL-6) (Figure 2D), which may also contribute to the LRNA9884 expression at certain degree. More importantly, we investigated the role of LRNA9884 in IL-1β driven renal inflammation via siRNA-mediated silencing (siLRNA9884) in mTECs *in vitro* (Figure 3A). Interestingly, both western blotting, real-time PCR and ELISA detected that silencing of LRNA9884 significantly suppressed the IL-1β-induced production of inflammatory cytokines including TNF-α, MCP-1 and IL-6 in mTECs compared to the nonsense-treated (NC) group (Figures 3B–D), indicating the importance of LRNA9884 in the progression of renal inflammation.

### LRNA9884 Is Essential for NF-κB Mediated Inflammatory Cytokines Production

**FIGURE 4:**
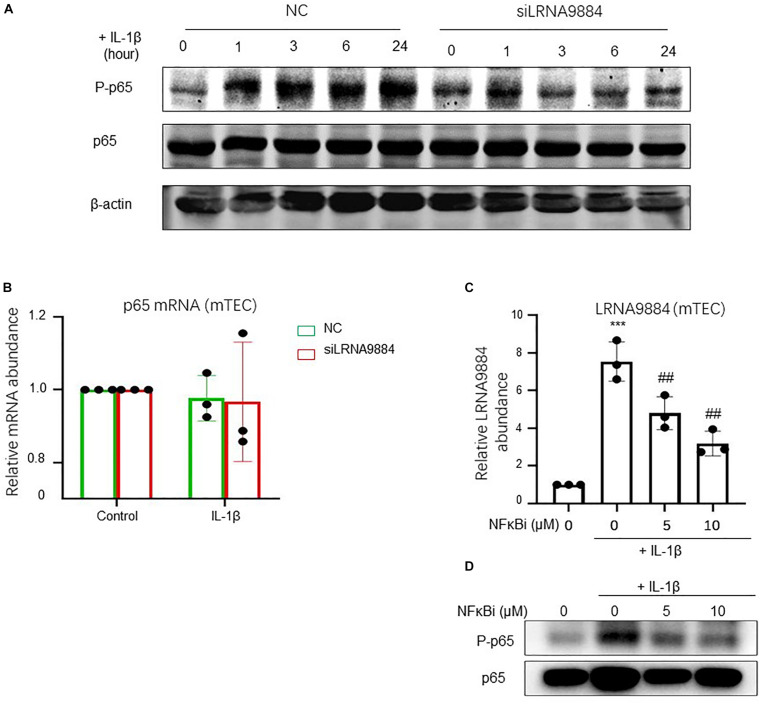
LRNA9884 regulates NF-κB mediated inflammatory actions. **(A)** Western blot shows phosphorylation and total NF-kB in NC and siLRNA9884 groups. **(B)** Real-time PCR shows total NF-kB expression (p65) in control and in IL-1β-induced mTECs groups. **(C)** Real-time PCR shows LRNA9884 expression after inhibition of NF-kB phosphorylation in mTECs in a dose-dependence. **(D)** Western blot shows phosphorylation and total NF-kB after inhibition of NF-kB phosphorylation in mTECs in a dose-dependence. Bars represent the mean ± SEM for at least three independent experiments. ***P < 0.001 vs. Control group, ^##^*P* < 0.01 vs. IL-1β-induced group.

NF-κB signaling is one of the important pathways for renal inflammation. Therefore, inactivation of the NF-κB pathway in AKI mice was studied by downregulating LRNA9884 production as well as using a NF-κB inhibitor (BAY 11-7082). Western blot showed that NF-κB phosphorylation was decreased after LRNA9884 expression levels were reduced (Figure 4A). Nevertheless, real-time PCR did not show changes in total NF-κB expression after silencing LRNA9884 (Figure 4B); hence LRNA9884 might only affect NF-κB phosphorylation but not its transcription. As a consequence, LRNA9884 expression was later studied following inhibition of NF-κB phosphorylation via real-time PCR. This analysis indicated that LRNA9884 expression was decreased by inhibition of NF-κB in a dose-dependent manner (*P* < 0.01) (Figure 4C). These findings suggested that there is a reciprocal regulation between LRNA9884 and NF-κB, in which NF-κB regulates LRNA9884 expression and LRNA9884 regulates NF-κB phosphorylation.

### LRNA9884 Promotes NF-κB-Induced MIF via Transcriptional Regulation

**FIGURE 5:**
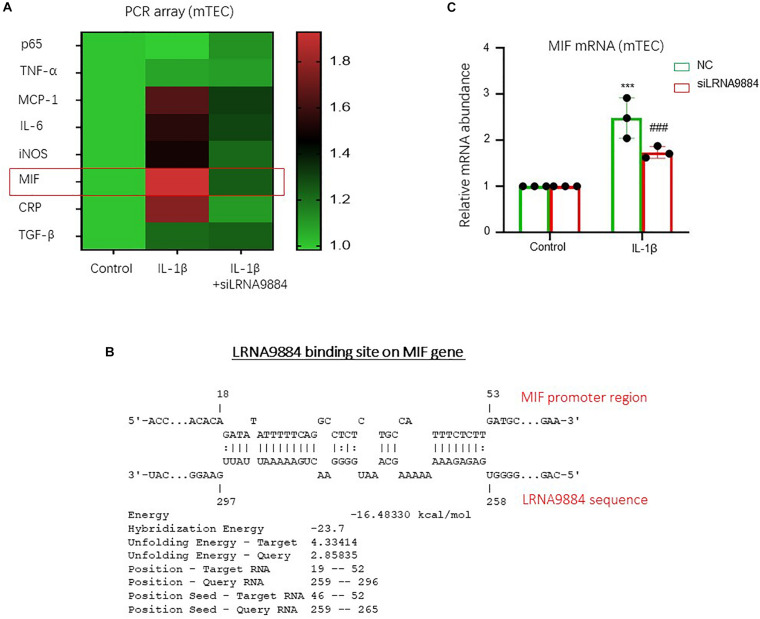
LRNA9884 regulates MIF at transcriptional level. **(A)** Real-time PCR array shows inflammatory markers expression after knockdown of LRNA9884 in mTEC. **(B)** Predicted binding site of LRNA9884 on the genomic sequence of MIF. **(C)** Real-time PCR results show MIF expression ratios in control and IL-1β mTECs groups. ***P < 0.001 vs. CTRL group, ^###^*P* < 0.001 vs. NC group.

**FIGURE 6:**
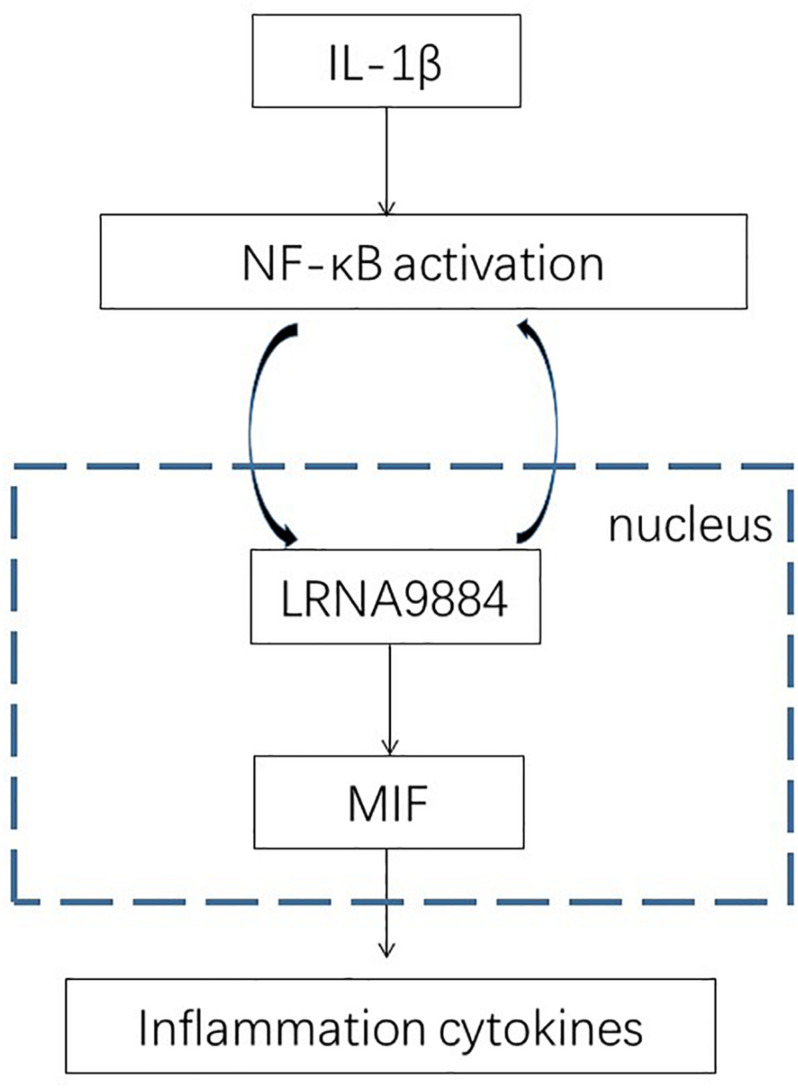
Schematic diagram of LRNA9884 in NF-kB/MIF axis regulation. In AKI, IL-1β triggers NF-kB signaling, which in turn upregulates LRNA9884 in the nucleus, and which binds on the promoter region of MIF genome therefore enhancing MIF transcription at genomic level. LRNA9884 will positively promote IL-1β/NF-κB signaling therefore further promoting the inflammatory response.

As previously described, LRNA9884 was involved in the downstream of NF-κB pathway (Zhang et al., 2019b), but the underlying mechanism is still largely unclear. Here, by conducting PCR array, we revealed that expression of an important pathogenic cytokine MIF was highly affected by the loss of LRNA9884 in mTECs under IL-1β-stimulation (Figure 5A). Interestingly, we found a direct LRNA9884 binding site on the promoter region of MIF using Freiburg RNA Tool (Figure 5B). We confirmed that MIF expression was significantly downregulated in renal tubular epithelial cells due to silencing of LRNA9884 (*P* < 0.001) (Figure 5C). Thus, LRNA9884 may promote NF-κB-mediated renal inflammation via enhancing MIF production at genomic level (Figure 6).

## Discussion

AKI can gradually progress into chronic renal failure due to persistent inflammation and progressive fibrosis, in which significant amounts of inflammatory and pro-inflammatory cytokines are released causing disease progression (Kinsey et al., 2008; Chawla et al., 2011). When tissue injury occurs in AKI, renal tubular epithelial cells injury, which leads to the release of pro-inflammatory factors from the cells to enhance the permeability of vascular endothelia. Then effector cells involved in immune responses such as macrophages extravasate to the injury site (Bonventre and Zuk, 2004; Friedewald and Rabb, 2004) to trigger the production of an inflammatory cascade, which further aggravates damage to the renal tubular epithelial cells. Therefore, effective inhibition of the production and secretion of various inflammatory factors in the initial stages of AKI may be the key to prevent AKI from progressing into chronic kidney disease.

Long non-coding RNAs have been suggested to play roles in the pathogenesis of various kidney diseases. For instance, LRNA9884 has been shown to be a promoting factor of diabetic kidney injury (Zhang et al., 2019b). In this study, it was found that LRNA9884 exerts a functional role in the pathogenesis of cisplatin-induced AKI. Cisplatin induced nephrotoxicity in mice are most prominently used to study AKI (Ramesh and Ranganathan, 2014). In our study, the expression of LRNA9884 was found to be significantly increased in renal tubular epithelial cells *in vivo*. Furthermore, *in vitro* analysis indicated that the increase in LRNA9884 expression occurs at early stages of AKI development since LRNA9884 expression incremented significantly after only 30 min upon IL-1β stimulation rather than cisplatin stimulation *in vitro* (Supplementary Figure 1). Increasing evidences showed IL-1β activation exacerbates cisplatin-induced AKI by activating inflammatory signals (Yan et al., 2017; Privratsky et al., 2018). Our results indicated LRNA9884 was relevant to inflammatory signaling. Thus, it was hypothesized that LRNA9884 might be involved in the inflammatory response of renal tubular epithelial cells during AKI. The present study revealed that LRNA9884 exhibits high expression levels in tubular epithelial cells after their stimulation with various inflammatory mediators. Meanwhile, we found IL-1β significantly up-regulated LRNA9884 expression compared other inflammatory stimulators *in vitro*. Moreover, it demonstrated that downregulation of LRNA9884 leads to declined production of various inflammatory mediators. Consequently, LRNA9884 may work as a potential regulator in the production of a cocktail of inflammatory factors. Lastly, it determined that LRNA9884 was confined within the nuclei of renal tubular epithelial cells rather than to their cytoplasm.

NF-κB is one of the most critical nuclear transcription factors present in virtually all animal cell types. NF-κB participates in classic inflammatory stimulation pathways, which are initiated by the activation of various external stimuli such as CRP, IL-1β, hypoxia induction, and among others. NF-κB phosphorylation initiates a response within 5 min of stimulation (Lawrence, 2009). Interestingly, we found that IL-1β might upregulate LRNA9884 via a P65-dependent mechanism. It is because the phosphorylated P65 can migrate into the nucleus and promote the transcription of LRNA9884. Thus, the AKI-included IL-1β can increase LRNA9884 expression in renal epithelial cells via activating P65 during renal inflammation. More importantly, inflammation-related RNA-sequence screening showed that the increased expression of MIF was LRNA9884-specific. MIF is an upstream pro-inflammatory factor and mainly functions as an activator of the inflammatory cascade, as a promoter of macrophage migration and as an initiator of T cell activation (Lan et al., 2018; Li et al., 2018a,b, 2019). Current study found that LRNA9884 has a binding motif in the MIF promoter region, thereby decreasing LRNA9884 levels may downregulate MIF expression. Our mechanistic study of LRNA9884 identified MIF might be as the direct downstream target of LRNA9884 in renal epithelial cells during IL-1β-mediated renal inflammation. LRNA9884 directly bind on the promoter region of MIF for enhancing its production from renal epithelial cells at transcriptional level, therefore further promoting the renal inflammation of the AKI-kidney. However, prospective studies are recommended to determine how LRNA9884 behaves and functions in human patients suffering from AKI. Similarly, they are suggested to conduct *in vivo* experiments and to use specific kidney silencing of LRNA9884.

In conclusion, the present study has provided evidence that injured renal tubular epithelial cells release inflammatory cytokines in AKI mice model, which upregulate LRNA9884 expression by activating phosphorylation of NF-κB pathway. This pathway subsequently upregulates MIF, triggering the production of a storm of other inflammatory response, which in turn aggravate renal tubular damage (Figure 6). These findings suggest that LRNA9884 has an adverse impact in AKI and targeting LRNA9884 represents a potential therapy for alleviating inflammation response in AKI kidneys.

## Data Availability Statement

The original contributions presented in the study are included in the article/Supplementary Material, further inquiries can be directed to the corresponding author/s.

## Ethics Statement

The animal study was reviewed and approved by Animal Experimentation Ethics Committee of The Chinese University of Hong Kong.

## Author Contributions

YZ and PT designed the research and performed cellular experiments. YN assisted animal experiments. YZ, PT, and YN analyzed results and wrote the manuscript. CG revised the manuscript. X-RH maintained animal availability. CY and H-YL guided and designed the manuscript. All authors contributed to the article and approved the submitted version.

## Conflict of Interest

The authors declare that the research was conducted in the absence of any commercial or financial relationships that could be construed as a potential conflict of interest.

## Footnotes

1 http://www.imagej.nih.gov/ij/

## Supplementary Material

Supplementary Figure 1 Cisplatin take no effect on LRNA9884 expression and silencing of LRNA9884 has no response to cisplatin induced apoptosis in mTECs.

Supplementary Figure 2 Quantitative analysis of western blot in mTECs.

Supplementary Table 1 Primer sequences (mouse).
