# Supplementary figures and images for: Long Non-coding RNA LRNA9884 Promotes Acute Kidney Injury via Regulating NF-kB-Mediated Transcriptional Activation of MIF
Source: Front Physiol. 2020 Oct 29;11:590027. doi: 10.3389/fphys.2020.590027 (PMC7658631; doi:10.3389/fphys.2020.590027)

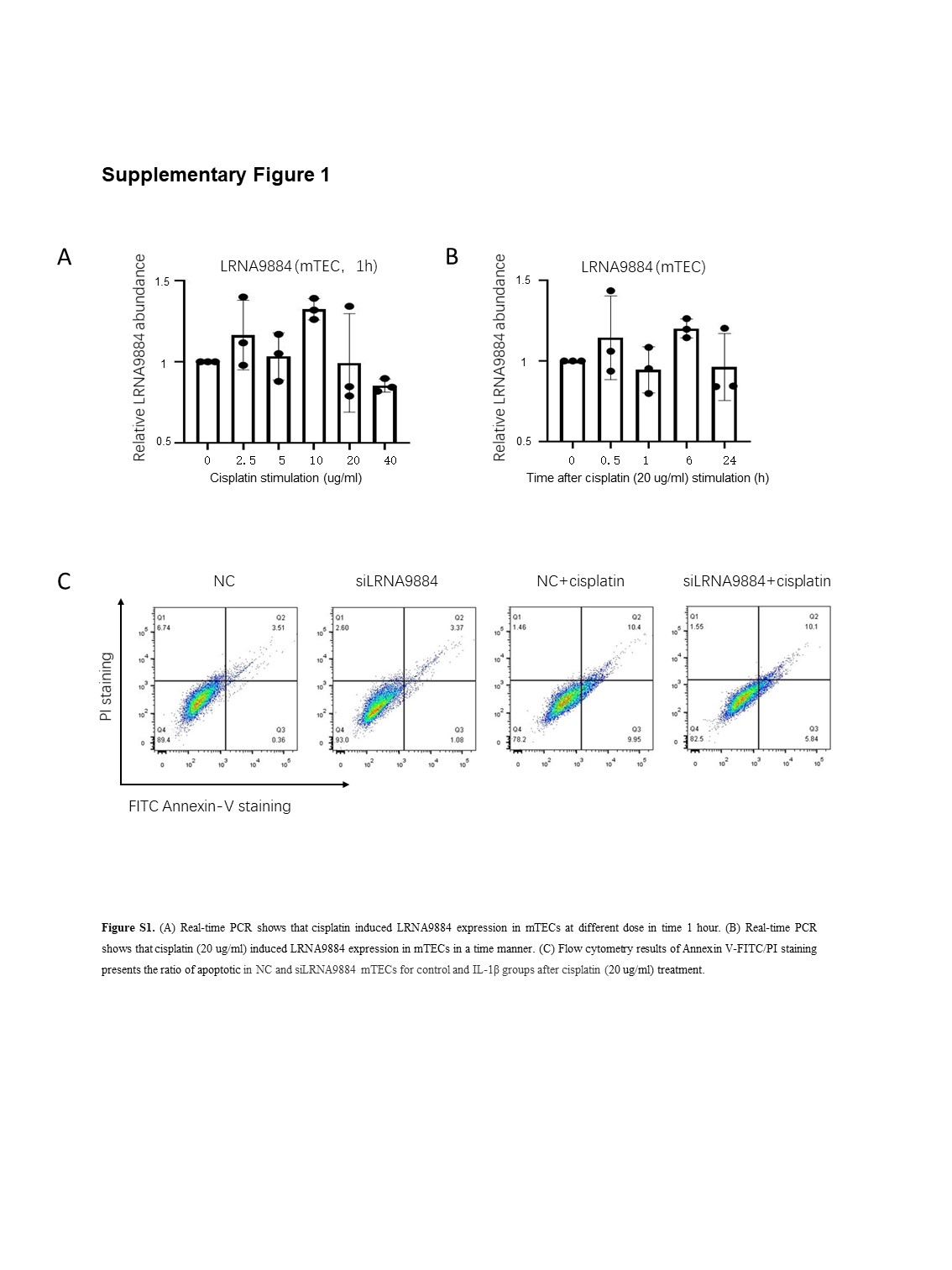

Supplement: Supplementary Figure 1 — Cisplatin take no effect on LRNA9884 expression and silencing of LRNA9884 has no response to cisplatin induced apoptosis in mTECs. [file Image_1.jpeg]

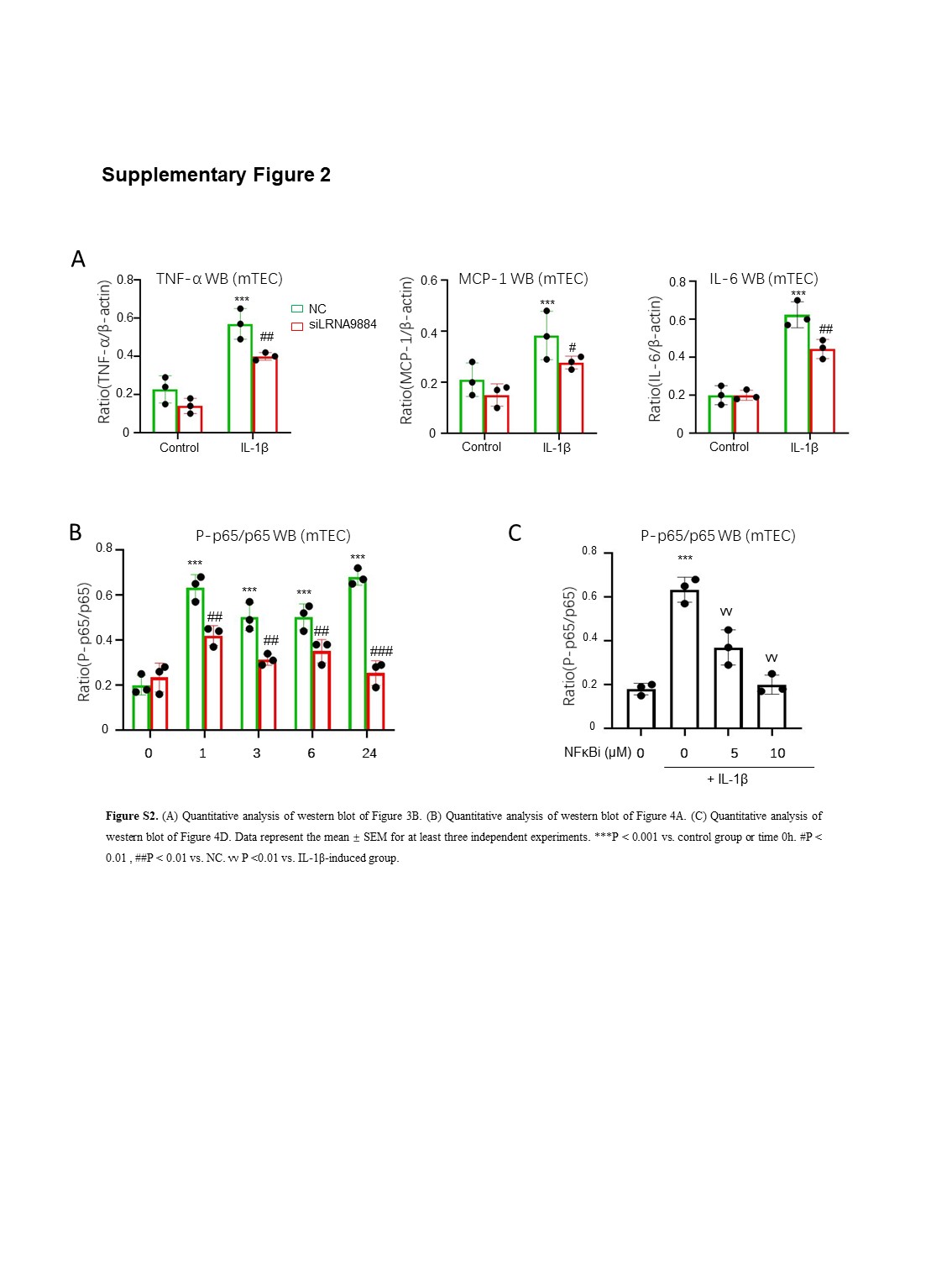

Supplement: Supplementary Figure 2 — Quantitative analysis of western blot in mTECs. [file Image_2.jpeg]

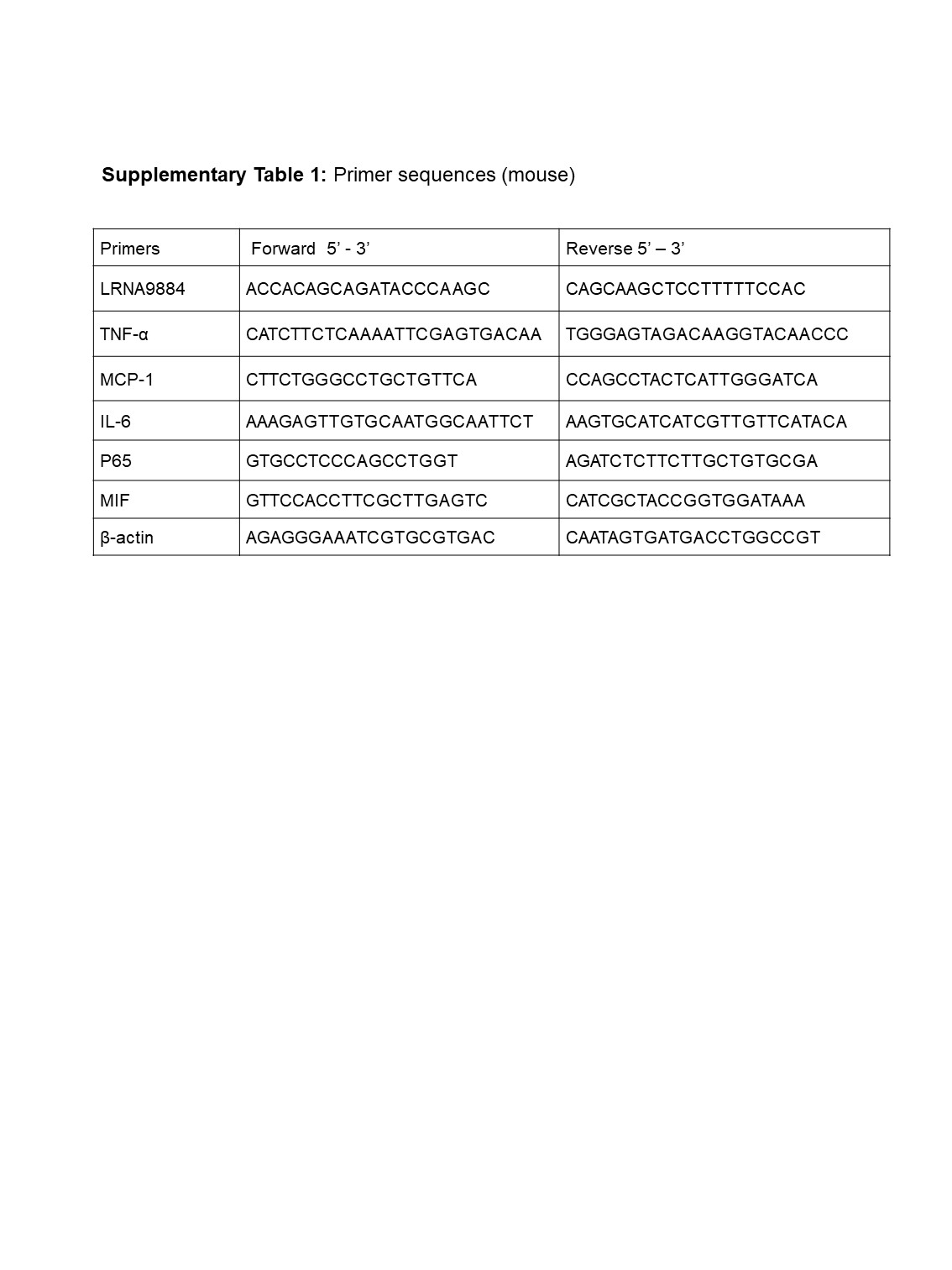

Supplement: Supplementary Table 1 — Primer sequences (mouse). [file Table_1.docx]
